# Apatinib plus retinoic acid as maintenance for children with relapsed stage 4 high-risk neuroblastoma: Two case reports

**DOI:** 10.1097/MD.0000000000020896

**Published:** 2020-06-26

**Authors:** Mei Jin, Dawei Zhang, Qian Zhao, Wen Zhao, Cheng Huang, Xisi Wang, Chao Duan, Yan Su, Xiaoli Ma

**Affiliations:** Beijing Key Laboratory of Pediatric Hematology Oncology; National Discipline of Pediatrics, Ministry of Education; MOE Key Laboratory of Major Diseases in Children; Hematology Oncology Center, Beijing Children's Hospital, Capital Medical University, National Center for Children's Health, Beijing, China.

**Keywords:** neuroblastoma, recurrence, treatment for recurrence

## Abstract

**Introduction:**

Metastatic neuroblastoma (NB) is an aggressive malignancy with a poor prognosis. Many patients present with relapsed high-risk NB after undergoing first-line treatment, and there is no standard therapy available in this setting.

**Patient concerns:**

The present study aimed to present the cases of 2 patients with recurrent high-risk NB.

**Diagnosis:**

Two children with International Neuroblastoma Stage System stage 4 high-risk NB chemotherapy. The disease recurrent after finishing the treatment.

**Interventions:**

Both patients (34 months old and 41 months old) experienced recurrence, received second-line treatment, and then received maintenance treatment using apatinib plus retinoic acid. The apatinib (10 mg/kg per day) and retinoic acid (160 mg/m^2^ per day) were administered on alternating 2-week cycles, which was continued for 1 year.

**Outcomes:**

The 2 patients had achieved complete response by the 1-year follow-up after starting apatinib plus retinoic acid, and did not experience any adverse drug reactions.

**Conclusion:**

The outcomes from these cases suggest that apatinib plus isotretinoin might be an option for maintenance therapy in patients with recurrent high-risk NB.

## 1 Introduction

Neuroblastoma (NB) is a rare malignant disease of the sympathetic nervous system that predominantly arises in children, with a median age at diagnosis of approximately 18 months.^[1]^ Despite intensive multimodality treatment, the 5-year event-free survival rate for children with high-risk NB remains <50%, and high-risk NB is responsible for 12% of pediatric cancer-related deaths.^[2,3]^ Furthermore, nearly 60% of patients who complete therapy will experience relapse of high-risk NB,^[4]^ and there is currently no standard therapy for relapsed/refractory high-risk NB. Therefore, effective strategies are urgently needed. This report describes our experience using apatinib plus retinoic acid as maintenance therapy for 2 patients with relapsed high-risk NB. Both patients responded well to the treatment.

## 2 Case presentation

The patients’ treatment was approved by the Beijing Children's Hospital Institutional Ethics Committee (No. 2017-Y-005). Informed consents were obtained from the parents or their guardians consent to the treatment and to the publication of the report in accordance with the Declaration of Helsinki.

### 2.1 Case 1

A 34-month-old boy was admitted to the hospital with a 7-month history of unexplained abdominal pain, and was diagnosed with International Neuroblastoma Staging System stage 4 high-risk NB. The chemotherapy involved the CAV regimen for cycles 1, 2, 4, and 6 (cyclophosphamide at 70 mg/kg on days 1–2, Adriamycin at 25 mg/m^2^ on days 1–3, and vincristine at 0.033 mg/kg on days 1–3), as well as the CVP regimen for cycles 3, 5, and 7 (cisplatin at 50 mg/m^2^ on days 1–4 and etoposide at 200 mg/m^2^ on days 1–3). After the first 4 cycles, the retroperitoneal tumor's size was decreased by 80% and there was complete response (CR) observed in the bone marrow and lymph nodes, which permitted primary tumor resection. After the 7 cycles of chemotherapy were completed, the patient underwent autologous stem cell transplantation and external beam radiation therapy. Isotretinoin therapy was maintained for 9 months.

**Figure 1:**
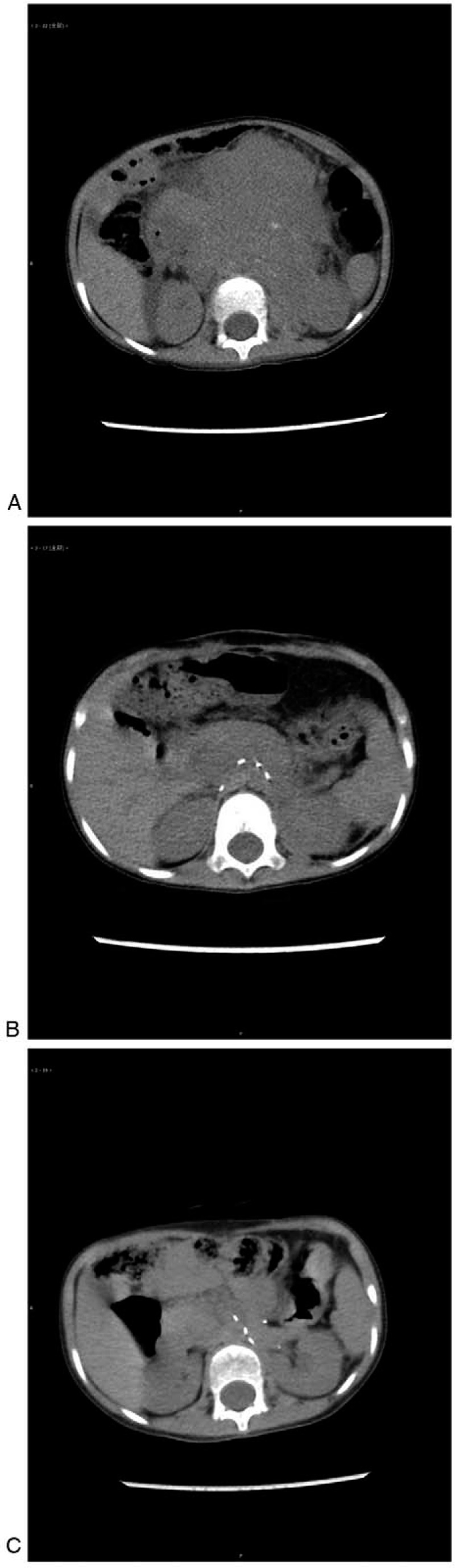
(A) Abnormal metabolism in the retroperitoneal tissue slightly to the left of the soft tissue mass with abnormally increased density and scattered calcification. (B) The patient achieved complete response after 9 mo of isotretinoin monotherapy. (C) Relapse of the celiac tumor.

Treatment responses were evaluated after cycle 2, cycle 4, and before starting maintenance treatment based on the Response Evaluation Criteria in Solid Tumors (version 1.1). The patient had achieved a CR before starting the maintenance treatment, but experienced relapse at 30 months after the diagnosis. Recurrence of the celiac tumor was detected via B-scan ultrasonography, although tumor maker levels were normal and bone marrow aspiration results were negative. A ^131^I-metaiodobenzylguanidine (^131^I-MIBG) scan revealed the relapsed celiac tumor and peritoneal lymph node metastasis. The patient underwent a second surgery and second-line chemotherapy using the TC regimen (topotecan and cyclophosphamide), CADO regimen (cyclophosphamide, vincristine, and doxorubicin), and CBVP regimen (carboplatin and etoposide). Maintenance therapy was subsequently performed using apatinib (10 mg/kg per day) and retinoic acid (160 mg/m^2^ per day) on alternating 2-week cycles, which was continued for 1 year. The patient completed follow-up evaluations every 6 weeks regarding tumor marker levels, bone marrow aspiration findings, and tumor imaging. The 1-year follow-up revealed that the patient had achieved CR (Fig. 1). Daily evaluations were performed to monitor the patient's temperature, blood pressure, and any skin rash at home, with weekly monitoring of urine, blood, and coagulation factors, as well as cardiac ultrasonography in local hospital. No adverse reactions occurred.

### 2.2 Case 2

**Figure 2:**
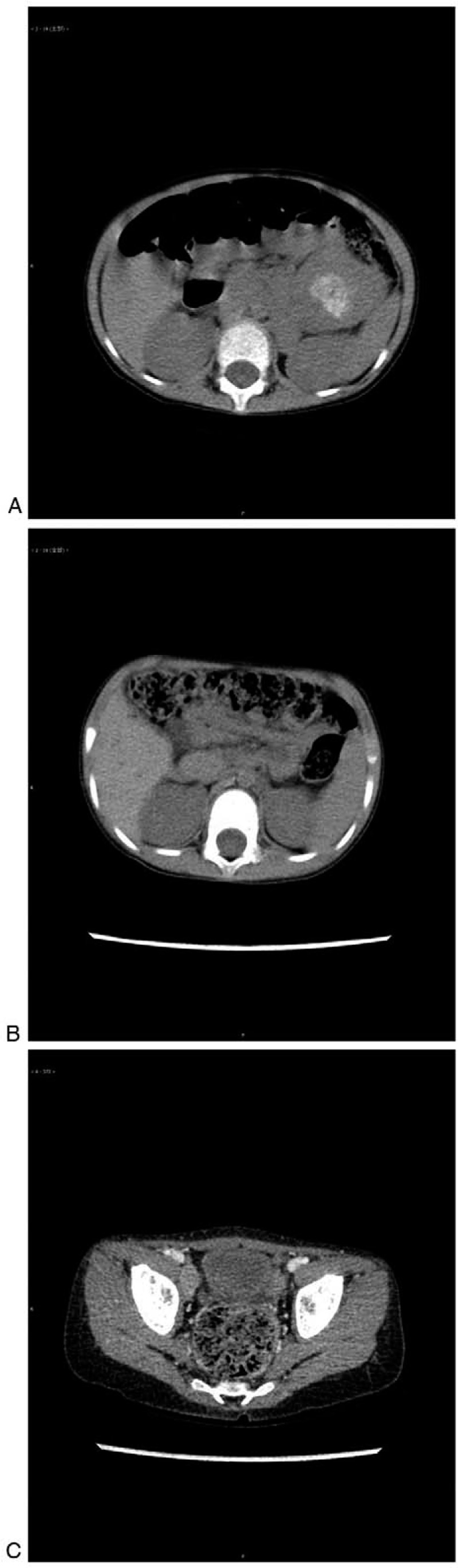
(A) The soft tissue mass with retroperitoneal lymph node metastasis and fusion between the left stomach and pancreas. (B) The patient achieved complete response after 9 mo of isotretinoin monotherapy. (C) Relapse of the right iliac fossa mass.

A 41-month-old boy was referred to the hospital with a 2-month history of joint pain. The primary tumor was retroperitoneal and involved the pancreas, and the diagnosis was International Neuroblastoma Stage System stage 4 high-risk NB. As in case 1, the patient underwent chemotherapy using the CAV and CVP regimens, followed by surgery, autologous stem cell transplantation, and external beam radiation therapy. The patient had achieved CR before starting the maintenance treatment. This patient also received isotretinoin as maintenance therapy for 9 months, but experienced relapse at 15 months after the diagnosis. A ^131^I-MIBG scan identified disease recurrence in the right pelvic cavity, left shoulder joint, upper right humerus, lower left femur, and upper tibia. Test results revealed mildly elevated neuron-specific enolase concentrations, but normal urine concentrations of homovanillic acid and vanillylmandelic acid. Bone marrow aspiration results were negative. As in case 1, the patient underwent second-line chemotherapy using the TC, CADO, and CBVP regimens. The soft tissue tumor remained in remission after 2 ^131^I-MIBG treatments at a 10-month interval. This patient also received maintenance therapy using apatinib (10 mg/kg per day) and retinoic acid (160 mg/m^2^ per day) on alternating 2-week cycles, which continued for 1 year. The 1-year follow-up revealed that the patient had achieved CR (Fig. 2). The patient was followed using the same protocol as for case 1, and no adverse reactions occurred.

The patients’ characteristics are provided in Table 1.

**Table 1:**
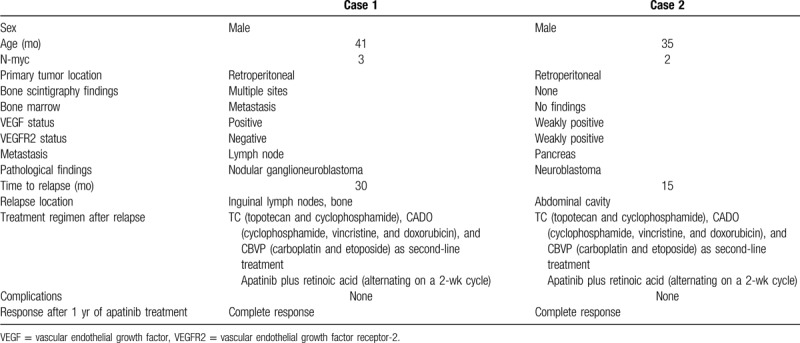
The patients’ characteristics.

## 3 Discussion

Many patients with high-risk NB experience complete clinical remission after receiving multimodal treatment, although relapse remains common. The 5-year overall survival rate is approximately 20% for patients after the first relapse of NB, although the International Society of Paediatric Oncology Europe Neuroblastoma group reported a progression-free survival rate of 5.7% and a median overall survival of 11.0 months for patients with relapsed disease.^[5]^ Patients with isolated relapse can undergo successful salvage treatment, which often involves surgery and/or focal radiation therapy. Additional chemotherapy is also needed for these patients. High-dose cyclophosphamide and topotecan plus vincristine has provided impressive results in this setting, including an overall response rate of 52% after the first relapse.^[6]^ The combination of ifosfamide, carboplatin, and etoposide is another a widely used option for children with relapsed NB, and a single-center study revealed disease regression in 14 of 17 patients (82%) with new relapse.^[7]^ A large phase II study evaluated 164 patients with relapsed or refractory NB who underwent ^131^I-MIBG treatment with autologous hematopoietic cell transfusion, with 36% of the patients exhibiting evidence of clinical response and 34% having stable disease for a median interval of 6 months.^[8]^ This result led to the inclusion of ^131^I-MIBG therapy in upfront consolidation treatment for children with high-risk NB. However, this strategy remains challenging because not all centers can administer high doses of radioactive iodine to pediatric patients, as well as the need for autologous stem cell rescue.

In the relapsed/refractory setting, immunotherapy targeting ganglioside G D_2_ (GD2) plus chemotherapy was recently found to provide a response in >50% of evaluated patients.^[9]^ Furthermore, the response rate was 61.5% among children with recurrent/refractory NB who received monoclonal anti-GD2 antibodies plus chemotherapy and natural killer cell infusions.^[10]^ However, anti-GD2 immunotherapy is not currently approved in China for patients with NB.

Vascular endothelial growth factor (VEGF) and angiogenesis play pivotal roles in tumor growth and metastasis in children. For example, children with cancer have increased circulating VEGF levels before they undergo primary tumor resection.^[11]^ Furthermore, VEGF is expressed in the primary tumor tissue of NB, with elevated expression being associated with invasion, metastasis, and recurrence.^[12]^ Apatinib is a small-molecule antiangiogenic agent that selectively inhibits the tyrosine kinase of vascular endothelial growth factor receptor-2 (VEGFR-2).^[13]^ Ultimately, this drug inhibits tumor angiogenesis through mechanisms involving VEGFR-2, blocking VEGF binding to VEGFR-2, inhibiting the phosphorylation of VEGFR-2, and downregulating the phosphorylation of downstream signal-regulated kinases. Moreover, apatinib inhibits the activity of VEGFR, platelet-derived growth factor, c-Kit, and c-Src, which are all tyrosine kinases that are thought to be associated with tumor development.^[14]^ Other studies have indicated that inclusion of apatinib in the treatment regimen could delay the development of resistance to conventional antitumor drugs by inhibiting the activity of ATP-binding cassette transporter subfamily B member 1 and subfamily G member 2.^[15,16]^

The first patient underwent surgical resection and chemotherapy after recurrence, which was followed by maintenance therapy using apatinib plus retinoic acid. The second patient experienced recurrence in multiple soft tissue and bone locations, which was treated using whole-body chemotherapy and ^131^I-MIBG therapy, and achieved remission of the soft tissue tumor. That patient also received maintenance treatment using apatinib plus retinoic acid. Both patients tolerated the treatments well and did not experience any obvious adverse events.

Approximately 63% of patients will present with relapsed disease at 6 to 18 months after diagnosis.^[17]^ Thus, effective maintenance therapy is a key strategy for treating NB. Maintenance treatment using apatinib plus retinoic acid appeared to have good tolerability and few side effects, which suggests it may be useful in this setting. However, a large well-designed clinical trial is needed to evaluate this strategy, and the treatment of patients with high-risk and relapsed NB remains challenging. Further studies are needed to identify biologically relevant pathways and novel therapies that harness the innate immune system to improve the prognosis of patients with NB.

## Author contributions

**Conceptualization:** Xiaoli Ma.

**Data curation:** Mei Jin, Qian Zhao, Cheng Huang.

**Formal analysis:** Mei Jin, Dawei Zhang, Cheng Huang.

**Investigation:** Cheng Huang.

**Methodology:** Mei Jin, Dawei Zhang, Chao Duan.

**Project administration:** Xiaoli Ma.

**Resources:** Dawei Zhang, Qian Zhao, Wen Zhao, Xisi Wang, Chao Duan.

**Software:** Qian Zhao, Wen Zhao, Xisi Wang, Chao Duan, Yan Su.

**Validation:** Dawei Zhang, Xisi Wang, Yan Su.

**Visualization:** Qian Zhao, Wen Zhao.

**Writing – original draft:** Mei Jin, Xiaoli Ma.

**Writing – review & editing:** Mei Jin, Dawei Zhang, Qian Zhao, Wen Zhao, Cheng Huang, Xisi Wang, Chao Duan, Yan Su, Xiaoli Ma.
